# Gene body methylation in cancer: molecular mechanisms and clinical applications

**DOI:** 10.1186/s13148-022-01382-9

**Published:** 2022-11-28

**Authors:** Qi Wang, Fei Xiong, Guanhua Wu, Wenzheng Liu, Junsheng Chen, Bing Wang, Yongjun Chen

**Affiliations:** Department of Biliary-Pancreatic Surgery, Tongji Hospital, Tongji Medical College, Huazhong University of Science and Technology, No. 1095 Jiefang Road, Wuhan, 430074 Hubei Province China

**Keywords:** Epigenetics, Gene body methylation, Histone methylation, Transcription regulation, Cancer

## Abstract

DNA methylation is an important epigenetic mechanism that regulates gene expression. To date, most DNA methylation studies have focussed on CpG islands in the gene promoter region, and the mechanism of methylation and the regulation of gene expression after methylation have been clearly elucidated. However, genome-wide methylation studies have shown that DNA methylation is widespread not only in promoters but also in gene bodies. Gene body methylation is widely involved in the expression regulation of many genes and is closely related to the occurrence and progression of malignant tumours. This review focusses on the formation of gene body methylation patterns, its regulation of transcription, and its relationship with tumours, providing clues to explore the mechanism of gene body methylation in regulating gene transcription and its significance and application in the field of oncology.

## Introduction

In the 1970s, Riggs AD and Holliday R proposed that methylation of the 5′-terminal cytosine (5′-C) of CpG dinucleotides is an important epigenetic marker of gene silencing that can cause changes in chromatin structure and DNA stability without altering the DNA sequence, thereby regulating gene expression [1, 2]. By the 1980s, it was found that CpG sites are heterogeneously distributed in the genome, with CpG sites tending to cluster in higher densities at promoter regions than throughout the rest of the genome, which has led to the definition of the term CpG islands (CGIs) [3], while the remaining 40–50% is scattered in intragenic regions and 30–40% in intergenic regions [4]. In the human genome, there are approximately 28,890 CGIs, most of which are located in the promoter regions of functional genes and are mostly unmethylated [3–5]. Approximately 9000 CGIs are located in the gene body regions, of which 30–40% are methylated [5–7].

With the rise of technologies such as genomic bisulfite sequencing, genome-wide DNA methylation mapping has been possible, and more extensive DNA methylation in the gene body region has attracted attention. Gene body methylation, which refers to the methylation of CpG sites in the transcribed regions of genes, including the methylation of scattered CpG sites and CGIs [8], was first described in Arabidopsis (Brassicaceae/Kale) in 2005 [9]. In 2006, the first genome-wide DNA methylation profile of Arabidopsis was described, showing that pericentromeric heterochromatin, repetitive sequences and regions producing small interfering RNAs around Arabidopsis thaliana mitoses are hypermethylated, and more than one-third of expressed genes are hypermethylated within transcribed regions, while only approximately 5% of genes exhibit methylation within promoter regions [10]. One year later, Zilberman discovered a significant correlation between gene body methylation and gene transcription in Arabidopsis [11]. Subsequent studies found that gene body methylation in animal genomes is similar to that in plants, preferentially occurring in exons of moderately, constitutively expressed and evolutionarily conserved housekeeping genes and having the same role in regulating transcription [12–15]. In human genomes, Rauch et al. and Maunakea et al. confirmed that methylated gene body regions correlated with higher levels of gene transcription [4, 5]. Besides, gene body methylation can affect processes such as histone modification [16], alternative splicing [17], and spurious transcription [18], discussed later in this review. At the same time, numerous studies have shown that abnormal methylation of gene bodies is closely related to gene expression, growth and differentiation, development, treatment and prognosis of tumours. In this review, we will discuss the distribution pattern of gene body methylation, its role and its relationship with tumours and explore the clinical application of gene body methylation and the urgent issues to be solved.

## Mechanism of gene body methylation patterning generation

### Active transcription promotes gene body methylation

**Fig. 1:**
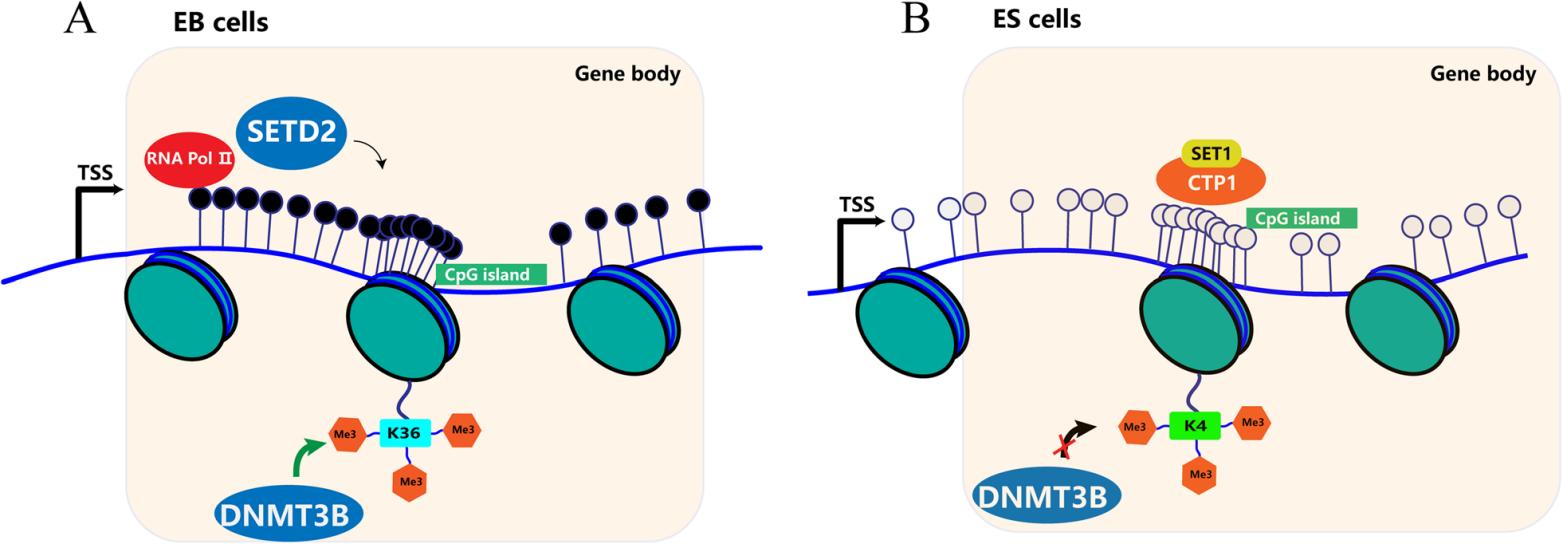
Effect of transcription on gene body methylation in RHPDF1 gene. **A** In embryoid body (EB) cells, RNA pol II recruits SETD2, methylating trimethylation of H3K36, in turn recruits DNMT3B leading to gene body methylation. **B** In ES cells, CGIs are bounding by CFP1, which mediates trimethylation of H3K4, blocking the recruitment of DNMTs

Although DNA methylation is commonly thought to lead to gene silencing, in the case of genomic methylation, it appears to be regulated by active transcription while affecting gene transcription. Following genetic and acquired genomic rearrangements, aberrant methylation of CGIs occurs when an unmethylated CGI is aberrantly recombined into another transcriptionally active gene in vivo [19, 20]. Thus, methylation of some CGIs may be a consequence rather than a cause of transcription. It has been shown that in the RHBDF1 gene without a promoter, the level of methylation of the gene body was significantly lower than in the RHBDF1 gene with a promoter. Besides, H3K36me3, a histone modification, is closely associated with gene body methylation through the recruitment of DNA methyltransferase 3B (DNMT 3B), and its distribution level can indirectly reflect gene body methylation levels [21]. It has been shown that H3K36me3 in the RHBDF1 gene was not enriched at the transcription start site and gradually increased throughout the gene body, reaching the maximum level near the transcription termination site. This phenomenon suggests that the methylation of CGIs within the RHBDF1 gene is dependent on promoter-mediated transcriptional processes. Overall, it has been shown that whether a specific alternative promoter or intergenic CGI becomes methylated depends on its chromosomal context and, in particular, on whether transcription passes through the CGI at some point [16] (Fig. 1A).

However, in ES cells, the CGIs through which transcription extends are not methylated. This is because, in ES cells, CGIs are usually bound by CpG binding factors such as CFP1, a component of the Set1 complex that mediates trimethylation of H3K4, a marker known to block the recruitment of DNMT 3A/3B/3L [22–25] (Fig. 1B). As cells differentiate, the H3K4me3 mark within the gene is gradually lost, and the transcriptional process gradually begins to mediate the methylation of CGIs within the gene [16].

In addition, while transcription can drive the methylation silencing of CGIs within a gene, it is not always sufficient to drive methylation of alternative promoter CGIs, which themselves act as strong initiators of transcription. Danuta et al. showed that the relative strengths of transcription initiation from the intragenic CGI and the promoter CGI of the gene within which it lies play an important role in determining the status of the intragenic CGI. There is speculation that CGIs that are longer [26] and have higher CpG density [27] may have stronger transcriptional activity. An alternative promoter CGI will not necessarily be silenced by elongating transcripts from a linked, but weaker, CGI promoter. These CGIs may perform additional biologically important functions.

### Interaction between gene body methylation and histone methylation

The majority of the promoter CGIs (~ 95%) and alternative promoter CGIs (~ 85%) are unmethylated (≤ 45% methylation). Their hypomethylated state is associated with H3K4me3 enrichment and lack of H3K36me3. In contrast, the majority (~ 65%) of gene body CGIs are DNA-methylated (≥ 55% methylation). The hypermethylated states of these CGIs are associated with traversing transcription elongation, H3K36me3 enrichment, and lack of H3K4me3 and H3K27me3 [16]. This phenomenon suggests a close link between gene body methylation and histone marks.

#### Roles of H3K4me3, H3K9me3 and H4K20me3 in DNA methylation

H3K4 is normally distributed on promoter CGIs and is regulated by the competitive binding of the CXXC domain of H3K4 methyltransferase and the ADD domain of DNMTs [22, 28]. Thus, DNA methylation and histone H3K4 methylation are negatively correlated. In the gene body, the vast majority of CGIs are hypermethylated, and thus, mostly lack H3K4me3 marks.

H3K9me3 binds primarily to heterochromatin and mediates DNA methylation of heterochromatin regions by the methyltransferase DNMT3A/B: 1. DNMT3A/B is recruited to heterochromatin by interacting with heterochromatin protein 1 (HP1) bound to H3K9me3 [29]; 2. DNMT3A/B also interacts directly with H3K9 methyltransferases. DNMT3A/B have been shown to interact with Suv39h1 [30] and Setdb1 [31] via their ADD domains. Furthermore, DNMT3A interacts with the euchromatin-associated H3K9 methyltransferase G9a/GLP via the chromatin domain protein MPP8 [32]. Additionally, a direct interaction between the C-terminal catalytic domains of DNMT3A/B and the ankyrin repeat domains of G9a, which are themselves able to bind to H3K9 methylation, has been shown to play roles in de novo methylation [33]. However, H3K9me is not clearly associated with gene body methylation, which was first found in plants [34]. Genome-wide methylation sequencing revealed that in suvh4, suvh5 and suvh6 mutant plants, gene body methylation levels were almost unaffected [35]. Therefore, the relationship between gene body methylation and H3K9me has been rarely studied.

H4K20me3 is a repressive mark found in constitutive heterochromatin and at imprinted genes where it is selectively enriched on the DNA-methylated allele [36–38]. In promoter regions, H4K20me3 represses gene expression as does DNA methylation [39], but the interaction between the two is not yet known. In heterochromatin regions, DNMT1 directly recognizes H4K20me3 through the first bromo-adjacent-homology domain, which in turn mediates the chromatin association and enzymatic activation of DNMT1 [40]. In the gene body regions, however, H3K20me3 is antagonistic to DNA methylation. The genetic regulator plant homologous structural domain finger protein 6 (Phf6) binds to the demethylated rDNA gene body and recruits the histone methyltransferase suv4-20h2 to establish the histone modification H4K20me3, which ultimately represses rDNA transcription. Gene body methylation prevents the recruitment of PHF6 and thus ensures rDNA transcription [41]. However, a direct interaction between H4K20me3 and gene body methylation has not been reported.

#### H3K36me3 and gene body methylation

H3K36me3 is mainly marked on the gene body and is formed by the histone methylation enzyme SETD2 and in conjunction with RNA Pol II catalyses the methylation of H3K36me0 [42], which is closely related to DNA de novo methylation. The de novo methylation of DNA is mainly initiated by the DNMT3 family [43], of which DNMT3B1 mainly binds to the gene body [21]. DNMT3 contains a PWWP domain that specifically recognizes H3K36me3 and is recruited to a highly abundant H3K36me3-labelled genomic region, causing the CpG site to undergo de novo methylation. Baubec et al. [21] knocked down the PWWP domain of DNMT3 or downregulated SETD2 in mouse embryonic stem (ES) cells and found that the accumulation of H3K36me3 was blocked and the level of gene body methylation decreased. This finding indicates that H3K36me3 plays an important role in recruiting DNMT3 and promoting gene body DNA methylation. H3K36me2 primarily recruits DNMT3A to catalyse and maintain DNA methylation in the intergenic region. Deletion of H3K36me2 results in redistribution of DNMT3A to H3K36me3-tagged gene bodies and reduces intergenic DNA methylation levels, suggesting that the PWWP structural domain of DNMT3A has dual recognition of H3K36me2/3 with a higher binding affinity towards H3K36me2 [44] and suggesting that posttranscriptional methylation of histone H3K36 plays a crucial role in the regulation of methylation levels in gene bodies.

#### H3K27me3 and gene body methylation

H3K27me3 is an epigenetic marker that inhibits the transcriptional elongation of RNA Pol II and promotes chromatin densification. H3K27me3 whole gene mapping shows that H3K27me3 is widely enriched in the gene body [45]. Removal of H3K27me3 from the gene body by JMJD3 demethylase promotes RNA Pol II elongation and activates the SETD2 complex, allowing H3K36me3 to accumulate in the gene body [46]. The relationship between H3K27 methylation and gene body methylation remains unclear, although H3K27me3 was shown to be antagonistic to H3K36me3. It has been found that H3K27me3 and DNA methylation occurred simultaneously in most regions of low CpG density in the gene body and only in CGIs were the two marks mutually exclusive. However, in DNMT knockout TKO cells, in addition to the high-density CpG regions where H3K27me3 markers were increased, large areas of H3K27me3 enrichment appeared in most regions of the genome [45]. This suggests that the two markers have an antagonistic effect on a larger scale of the genome in addition to a local antagonistic effect in the high-density CpG region. However, it has also been shown that gene body methylation coexists with H3K27me3 to maintain the chromatin dense state and repress transcription [47]. In conclusion, the relationship and interaction mechanism between the two have not been elucidated, and further experimental studies are still needed.

In conclusion, there is an inextricable relationship between histone modifications and DNA methylation, with histone modifications regulating DNA methylation and DNA methylation influencing histone modifications and acting further through them.

## Functions of gene body methylation

DNA methylation near the transcription start site usually has a repressive effect on gene expression, but methylation of the gene body has the opposite effect [48–52]. This phenomenon is known as the “DNA methylation paradox” [53], and recent studies have revealed a more complex relationship between genomic methylation and transcription.

### Preventing spurious transcription initiation

**Fig. 2:**
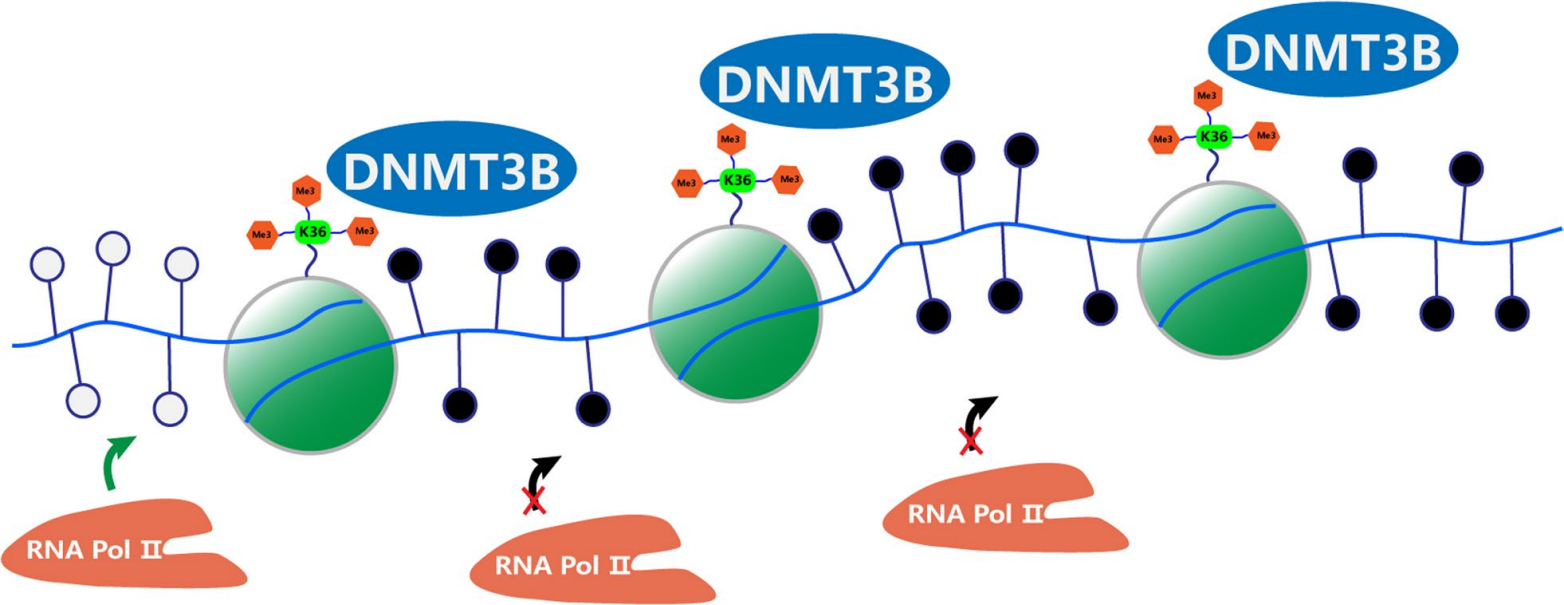
Preventing spurious transcription initiation. H3K36me3 recruits DNMT3B, methylating the CGIs, which block the binding of RNA Pol II

Spurious transcription is the process by which RNA polymerase binds to an ectopic promoter rather than a normal promoter to produce an abnormal transcript [54]. Some of the abnormal transcripts are polyadenylated and stabilized to produce abnormal proteins, which in turn affect molecular mechanisms, including gene expression regulation [55], miRNA targeting [56] and truncated protein generation [57, 58], leading to tumour cell heterogeneity and tumour susceptibility [18]. It has been found that Dnmt3b anchoring and binding to H3K36me3 in the gene body region inhibits RNA Pol II from binding to cryptic transcription initiation downstream of normal promoters by methylating the bases at abnormal transcription start sites [18]. Lorincz et al. [59] found that the density of RNA Pol II in the methylated region of the gene body is significantly reduced. Rapelli et al. [18] also found that in Dnmt3b−/− ES cells, a significant amount of RNA is transcribed from exons of the gene body. These findings confirm, both positively and negatively, that gene body methylation plays an important role in maintaining transcriptional fidelity, which prevents abnormal intragenic transcription by blocking the binding of RNA Pol II to ectopic transcription initiation within the gene body (Fig. 2).

### Inhibition of transcriptional elongation

Acetylation of the N-terminal lysine of histones is closely associated with transcriptional activation, and this modification neutralizes the positive charge of the lysine, thereby inducing a change in nucleosome conformation and making it easier for nucleosomes to participate in transcription [60, 61]. Gene body methylation induces hypoacetylation of histone lysine, promotes the formation of dense chromatin structures, prevents binding of TFs, and reduces the efficiency of RNA Pol II elongation [59].

### Regulating alternative splicing

In eukaryotes, pre-messenger RNAs (pre-mRNAs) undergo splicing, a process of maturation by which large intervening sequences (introns) are removed, leaving a mature transcript composed only of exons spliced together [17]. This process serves as a crucial regulatory step of gene expression and transcriptome diversification, as almost all genes produce alternative transcripts with differing exon compositions [62]. A higher level of methylation was found in the region near the exon start site and the termination site, suggesting that DNA methylation is involved in the process of exon alternative splicing. The higher the exon methylation density (total exon methylation level divided by the length of the corresponding exon), the higher the level of inclusion and expression of the exons in the transcript [63].

**Fig. 3:**
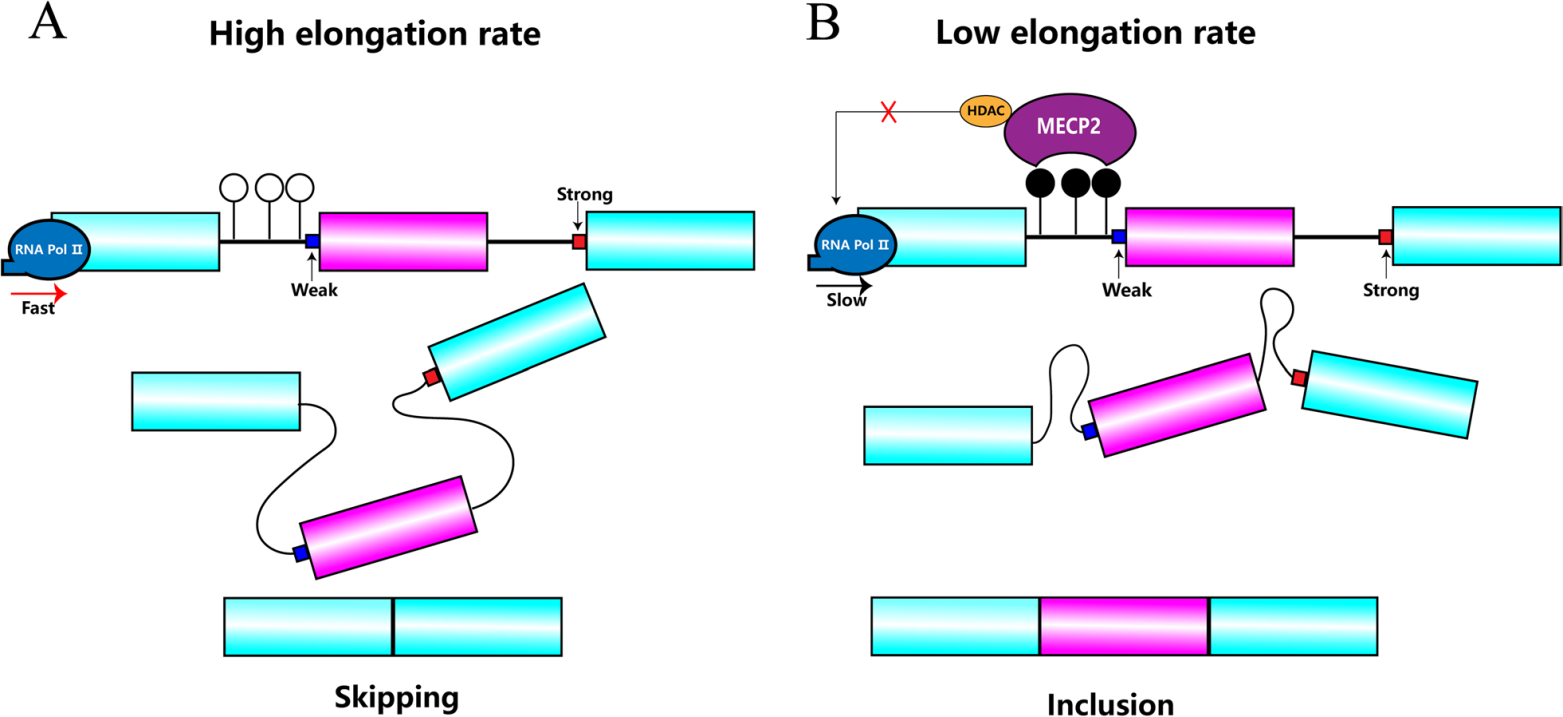
Gene body methylation and alternative splicing. **A** When the CGIs are unmethylated, the RNA pol II moves fast and weak splice sites are ignored. **B** When the CGIs are methylated and bound by MECP2, the RNA pol II moves slow and weak splice sites can be recognized

Two mechanisms that are not mutually exclusive have been proposed to explain how epigenetic modifications work: the kinetic model and the recruitment model. In the kinetic model, epigenetic modifications influence the kinetics of transcriptional elongation, which in turn affects splicing. This includes affecting the pace at which splice sites and regulatory sequences emerge in nascent precursor mRNAs during transcription, the rate of movement of RNA Pol II, and the open state of chromatin [64]. During transcription, when RNA Pol II moves faster, weaker splice sites are skipped, and exons at the original weak sites are skipped (Fig. 3A). When RNA Pol II moves more slowly, there is sufficient quantity and time to recognize the weaker splice sites, thus facilitating the inclusion of the corresponding exons (Fig. 3B). For example, the DNA-binding protein CTCF, which recognizes a specific unmethylated DNA sequence, binds to DNA and reduces the transcriptional elongation rate of RNA Pol II, thus facilitating exon inclusion; when the DNA is methylated, CTCF is unable to bind to it, the transcriptional elongation rate of RNA Pol II becomes faster, and exons are skipped [65]. In addition, the DNA-binding protein MECP2 recognizes and binds to methylated DNA and then recruits histone deacetylases, which reduce the rate of RNA Pol II movement and thus facilitate exon inclusion [66]. In the recruitment model, splicing regulation is accomplished through the binding of epigenetic modification sites by adaptor proteins and the recruitment of splicing factors [67]. For example, histone H3K36me3 can recruit splicing factors PTB [68] and SRSF1 [69] to regulate alternative splicing. In addition, DNA methylation can cause histone H3K9me3 to bind to heterochromatin protein 1 (HP1), which recruits the splicing factor serine- and arginine-rich splicing factor 3 (SRSF3) to the methylated region of the gene to achieve alternative splicing; when HP1 binds to an alternative exon, it leads to exon skipping, and when HP1 binds to an intron immediately upstream of the alternative exon, it promotes exon containment [70].

### Enhancer methylation

It has been found that demethylation of the genome results in the appearance of many new active enhancers, evenly distributed across gene bodies, and mediates the upregulation of gene expression, suggesting that gene body methylation silences enhancer function [71]. This is mainly associated with reduced chromatin accessibility and reduced binding of transcription factors (TF) [72]. The level of methylation at TF binding sites is negatively correlated with TF expression, and TF binding induces demethylation of binding sites, so enhancer methylation may be a consequence of reduced TF expression [72]. For example, the binding of glucocorticoid receptors to tissue-specific enhancers induces DNA demethylation, leading to enhancer activation [73]. However, the relationship between DNA methylation and enhancer activity remains incompletely understood, and it has been shown that high levels of DNA methylation coexist with H3K27ac in conventional and super-enhancers and that these regions are generally located outside of the TF binding site and therefore do not inhibit enhancer activity but rather stabilize DNA and define demethylated TF binding sites [74]. In addition, bioinformatic analyses have shown that CGIs themselves exhibit enhancer histone modifications and show greater transcription factor binding [75]. Such signatures have been identified at a gene body CGI within Kdm6b, which exhibits H3K4me1 and loops to the promoter CGI to enhance Kdm6b expression [76]. In addition, the distribution of enhancer CGIs plays an important role in enhancer function; enhancers enriched in CGIs (i) are more highly conserved, (ii) are more highly enriched in R loops and 3D genomic contacts, (iii) on average provide stronger signals in functional assays, and (iv) are prone to hypermethylation in cancer [77].

### Gene body methylation and cell differentiation

Recent studies have revealed that many genes specifically expressed in tissues and involved in encoding key regulators of morphogenesis and organ development contain genomic CGIs, which may function as cis-acting elements controlling tissue-specific transcription [78]. By comparing human embryonic stem cells with differentiated cells, such as human neural progenitors and fibroblasts, significant changes in gene body CGI modifications were found to correlate with differentiation-induced gene expression. In embryonic stem cells, intragenic CGIs are usually bivalently modified by H3K4me3 and H3K27me3 and are not methylated. In differentiated cell gene bodies, however, there is a lack of bivalently modified CGIs, and some CGIs are monovalently modified by high levels of H3K27me3 without methylation; approximately one-third are hypermethylated, which is associated with gene activation, and these hypermethylated genes play an important role in growth and development [78]. This is associated with gene activation, and these hypermethylated genes play important roles in growth and development. For example, the differentiation of pluripotent stem cells into neuronal precursor cells is mediated by the binding of the proneural factor NEUROD1 (a transcription factor that induces differentiation into neuronal cells) to hypermethylated regions in specific gene bodies [79]. These results suggest that during differentiation, gene body CGIs regulate gene expression by converting bivalent histone modification to DNA methylation at poised developmental genes or by gaining H3K27me3 marks to further silence genes, the expression of which is not needed. Cell-type-specifically expressed transcription factors and their target genes may take advantage of epigenetic modifications of gene body CGIs to generate a spectrum of differentiation from embryonic stem cells [78]. It is thus clear that gene body methylation is closely linked to cell development and differentiation and plays an important developmental regulatory role.

Gene body methylation also plays an important role in the aberrant differentiation of tumour cells. In prostate cancer cells, DNMT1 is reduced to promote epithelial–mesenchymal transition (EMT) and conversion to tumour stem cells by promoting PKCα expression, which in turn promotes prostate cancer growth and metastasis [80]. In triple-negative breast cancer (TNBC), DNMT1 promotes the EMT required for metastasis by regulating the methylation levels of epithelial markers [81]. Another study found that HCT116 human colorectal cancer cells inactivated with DNMT1 and DNMT3B (DKO cells) showed increased expression of mesenchymal markers and decreased expression of epithelial markers, with marked changes in cell morphology and epithelial–mesenchymal transition. The splicing factors ESRP1, RBFOX2, and NBML1, which affect the composition of CD44 exons, were also downregulated in DKO cells, suggesting that the development of EMT is associated with DNA methylation and its resultant CD44 alternative exon skipping [57]. These results suggest that DNA methyltransferase deficiency plays an important role in EMT and has a significant impact on tumour hypofractionation and growth invasion. However, this contradicts the role of the antitumor DNA methyltransferase inhibitor drugs, and the current study does not clearly identify the region of genomic demethylation. Further studies are needed to investigate the mechanism of how demethylation induces tumour dedifferentiation.

## Gene body methylation and cancer

### Tumour repeat element hypomethylation

The majority of CpG sites are found in repeat elements (REs) that occupy two-thirds of the genome, including interspersed repeats and tandem repeats [82, 83]. Repeated elements play a role in chromosome structure formation [84], gene transcription [85, 86] and nuclear structure [86]. In tumours, more than 2/3 of tumour genome-wide hypomethylation occurs in REs [87] (Table 1). REs hypomethylation can lead to reduced resistance of the genome to DNA damage [88], affect the stability of the mitogen and surrounding regions [89] and promote chromosomal rearrangements [90]. This can affect genomic stability and even induce oncogene activation. Furthermore, RE hypomethylation seems to be a unique feature of tumours that is not found in precancerous lesions or peripheral blood [91, 92] and therefore has potential use in cancer diagnosis and prognosis. However, the detailed mechanism by which RE methylation affects mitophagy function remains an urgent question. In addition, RE hypomethylation is also associated with hereditary diseases, neurodevelopmental, neurodegenerative, and neuropsychiatric disorders as well as ageing [93].

**Table 1:** Effect of REs hypomethylation on tumours

REs	Tumours	Effect	References
LINE-1	Lung cancer	Cancer stage and prognosis	[94]
Alu	Thyroid cancer	Metastasis and prognosis	[95]
LINE and Alu Yb8	Bladder cancer	Cancer stage or disease outcome	[96]
Sat2, Satα, and Alu	Liver cancer	Gain of chromosome 1q and diagnosis of cancer	[89, 97]
Satα	Nephroblastoma	Loss of chromosome 16q Gain of chromosome 1q	[98]
Sat2, Satα, LINE1 and Alu	Ovarian epithelial tumour	Prognosis and biomarkers	[99–101]
LINE-1and Alu	Breast cancer	Risk, progression, and type of cancer	[102, 103]
LINE-1 and Alu	Gastrointestinal tumours	Cancer stage and prognosis	[104–106]

### Gene body methylation and tumorigenesis

Huang et al. found that intestinal epithelial saprophytic cells exhibit aberrant hypermethylation in the early stages, but global gene body hypomethylation occurs in the transition from intestinal epithelial saprophytic cells to cancer cells [107]. This suggests that gene body methylation plays an important role in the development of tumours. In normal cells, METTL7A is a key protein for lipid metabolism and the generation of functional organelles. EZH2 interacts with the CpG sites of the METTL7A gene body in thyroid cancer, recruiting MBD2 and rejecting RNA pol II, resulting in METTL7A silencing, which in turn mediates abnormal tumour metabolism [108]. Significant gene body CGI hypomethylation was observed in hepatitis B virus X (HBx)-infected livers. HBx promotes hepatocarcinogenesis by recruiting histone deacetylase 1 (HDAC1) to repress the Dnmt3L and Dnmt3a promoters, inducing gene body CGI hypomethylation and downregulation of associated developmental regulators [109]. Lai et al. found that in lymphoma cells with high expression of BCL6, CGI within the first intron of the BCL6 locus was hypermethylated. The transcription factor CTCF, which blocks the enhancer, is usually bound to unmethylated DNA. During lymphomagenesis, hypermethylation of the first intron DNA blocks CTCF binding, thereby allowing the enhancer to exert its effects and increase BCL6 expression, promoting the development of lymphoma [110]. Cheung et al. [111] found that in testicular malignancies, hypermethylation of an intron region spanning miR-199a resulted in downregulation of its expression, which in turn led to abnormal upregulation of the expression of its target protein podocalyxin-like protein 1 (PODXL), affecting the aggressive behaviour of testicular tumours. The inositol triphosphate 3-kinase A gene (ITPKA) is a potentially oncogenic gene that is upregulated in a variety of cancers. Wang et al. found that the gene body CGI of ITPKA competes with the promoter to bind the SP1 transcription factor and that methylation of the gene body promotes SP1 binding to the promoter and thus ITPKA expression. In lung cancer, the methylation of ITPKA occurs in in situ carcinoma and increases with the level of invasion [112]. In addition, HOX transcript antisense RNA (HOTAIR), a widely studied long-stranded noncoding RNA (lncRNA), was found to be significantly overexpressed in different cancer types with different genetic profiles. It interacts with microRNAs (miRNAs) as a competing RNA to induce dysregulation of key genes, thereby driving important cancer phenotypes. In a variety of tumours, CDK9-mediated phosphorylation of RNA Pol II Ser2 and MLL1-mediated regulation of H3K4me3 allow for hypermethylation of CGIs in exons of the HOTAIR gene, facilitating the transcriptional elongation process and promoting HOTAIR overexpression, which in turn mediates tumour development [113]. McGuire et al. found that lymphocyte-specific gene CARD11 gene body methylation levels were significantly and positively correlated with expression levels, were highly expressed in renal cell carcinoma and lung adenocarcinoma, and activated the mTOR pathway to inhibit cellular autophagy, thereby promoting tumour development [114] (Table 2).

**Table 2:** Gene body methylation and tumorigenesis

Type of cancer	Gene	Functions	References
Thyroid cancer	METTL7A	Metabolic abnormalities	[108]
Hepatocarcinoma	HBx	Dysregulation of gene expression	[109]
Lymphoma	BCL6	Oncogene overexpression	[110]
Testicular cancer	miR-199a	Promotes tumour invasion	[111]
Lung cancer	ITPKA	Promotes tumour invasion	[112]
Renal cell carcinoma and lung adenocarcinoma	CARD11	Inhibits autophagy	[114]

In summary, gene body methylation, similar to promoter DNA methylation, has been shown to play an important role in the occurrence and development of tumours. However, unlike promoter DNA methylation, which mediates tumour development by suppressing oncogene expression, gene body methylation plays a complex role, and the relevant research results are relatively one-sided. As a result, the role of gene body methylation in different tumours still needs to be analysed in specific genes. It is expected that with the gradual exploration of gene body methylation, there will be a complete and accurate theory to explain its abnormal distribution and mechanism of action in tumours.

### Gene body methylation and tumour therapy

There are two main types of DNMT inhibitors. One is a nucleotide analogue that inhibits its activity by replacing cytosine during DNA replication and forming a covalent bond with DNMT. Currently, nucleotide analogues of DNMT inhibitors are approved for use by the U.S. Food and Drug Administration (FDA) and show great clinical value in haematologic and solid tumours [115, 116]. Previously, DNA methylation inhibitors were used to reactivate the promoters of methylation-silenced tumour suppressor genes, but these drugs also have a demethylating effect on gene body sequences. Due to the positive correlation between gene body methylation levels and expression levels, gene body demethylation of oncogenes and aberrant metabolic genes in tumours may mitigate their overexpression and thus attenuate their positive effects on tumour development. For example, DNA demethylation agents acting on the gene body of the oncogene C-MYC have been found to have a potential role in attenuating its oncogenic effects [55]. In addition, treatment of cells with 5-aza-CdR revealed that the duration of gene body demethylation nearly overlapped with the time of significant inhibition of cell growth, suggesting that changes in DNA methylation may determine the rate of recovery of cell growth. Genes downregulated after demethylation were upregulated with subsequent DNA remethylation, which was dependent on DNMT3B and local chromatin structure. The combination of DNA methylation inhibitors with DNMT3B inhibitors is therefore expected to prolong the antitumour effect and provides an important direction for future research [55].

Another DNMT inhibitor, which is still in the research stage, is a non-nucleotide analogue that binds directly to the region on DNMT where its methylation acts to inhibit its function [117]. Recent studies have found that 18β-glycyrrhetinic acid (GRA) has the same demethylating effect as DNMT inhibitors in gastric cancer tissues. The expression level of ATP4a, a differentiation-related gene, was significantly downregulated in gastric cancer tissues, and the methylation level of the gene body was negatively correlated with the expression of ATP4a. GRA had a demethylation effect on ATP4a in vivo and in vitro, activating the expression of ATP4a and inhibiting the occurrence of gastric cancer, suggesting that GRA is an effective demethylating agent with certain clinical application prospects for the treatment of gastric cancer [118].

DNMT inhibitors, as widely used antitumor drugs at present, have not received much attention for their demethylation effects on gene body regions. The above studies have provided clues to the role of DNMT inhibitors in gene body methylation, which may inhibit abnormally overexpressed genes in tumours and have a certain therapeutic effect on tumours. Therefore, future studies can investigate gene body methylation levels as a new therapeutic target, focussing on the role and effects of DNMT inhibitors on gene body methylation and identifying combination drugs or antitumor drugs that specifically target gene body methylation.

### Gene body methylation and prognostic assessment of tumours

GenE body methylation abnormalities, as a cause of tumorigenesis, are of great significance as prognostic biomarkers in tumours. There is currently widespread interest in comparing the impact of methylation differences on survival through the analysis of biological information and finding corresponding biomarkers.

In head and neck squamous cell carcinoma, lincRNA C5orf66-AS1 methylation levels are significantly negatively correlated with overall survival, which is a promising prognostic biomarker in head and neck squamous cell carcinoma and can be used to identify HPV-negative patients at risk of recurrence and metastasis [119]. In glioblastoma, the ZMIZ1 gene is a driver of tumour cell migration. ZMIZ1 gene body hypermethylation inhibits tumour cell migration by regulating variable splicing and reducing the expression level of transcripts with higher potency, so patients with ZMIZI gene body hypermethylation tend to have a better prognosis [120]. In lung cancer, 260 exons were found to have differential methylation levels and expression levels in lung cancer tissue compared to normal lung tissue. Among these markers, exon 10 methylation markers were able to significantly differentiate between high-risk and low-risk patients, with promising clinical applications in the prognosis and surgical management of lung cancer [63]. In myelodysplastic syndromes, 57% of patients had DNMT3A aberrant gene body hypomethylation. The multifactorial analysis confirmed that DNMT3A methylation status was an independent prognostic factor in patients with MDS [121].

In gastrointestinal tumours, Liu et al. identified methylation changes in the GFRA1, SRF and ZNF382 genes as potential synergistic biomarkers for predicting gastric cancer metastasis [122]. Debernardi et al. found that the long interspersed nuclear elements 1, located in the second intron of MET oncogene (LINE1-MET), hypomethylation is an important feature of early-onset colorectal cancer (CRC), that LINE1-MET hypomethylation in colorectal adenomas is associated with a high risk of developing colorectal cancer, and that LINE1-MET methylation levels are of great significance for prognostic assessment of patients after surgical resection of polyps [104]. Triple repair nucleic acid exonuclease 2 (TREX2) has a DNA repair function and may exert a tumour suppressive effect. Weigel et al. found that TREX2 gene body hypomethylation resulted in enhancer activity of the TREX2 motif, which promoted TREX2 expression and thus exerted a tumour suppressive effect. TREX2 hypomethylation was strongly associated with prolonged overall survival in laryngeal and colorectal cancers [123]. Bormann et al. found that amphiregulin (AREG) expression was positively correlated with increased progression-free survival and overall survival. Further studies found that AREG gene body methylation levels were significantly negatively correlated with AREG expression levels and therefore may also have some relevance to response after epidermal growth factor receptor (EGFR) therapy. However, a retrospective study found that AREG gene body methylation levels are not as predictive as AREG mRNA levels and cannot yet be used as a clinically reliable biomarker, but they still provide an important clue for further research [124]. In addition, in patients with cholangiocarcinoma, patients with higher methylation levels of PITX2-adjacent noncoding RNAs had relatively longer survival and could be an independent prognostic factor [125] (Table 3).

**Table 3:** Gene body methylation and prognostic assessment of tumours

Tumour type	Gene	Correlation of gene body methylation levels and survival	References
Head and neck squamous cell carcinoma	LincRNA C5orf66-AS1	Negative correlation	[119]
Glioblastoma	ZMIZI	Positive correlation	[120]
MDS	DNMT3A	Positive correlation	[121]
Colorectal cancer	LINE-1	Negative correlation	[104]
	AREG		[124]
Laryngeal carcinoma	TREX2	Negative correlation	[123]
Cholangiocarcinoma	PITX2	Positive correlation	[125]

The above studies show that gene body methylation provides a clear indication of prognosis for a variety of tumours and can be an important supplement for tumour prognosis judgement. Unfortunately, the relationship between gene body methylation and tumour prognosis is still at the level of bioinformatics analysis, and its specific mechanism has not been fully explained, which makes gene body methylation not convincing enough as a biomarker and cannot be widely used in different tumours. Future research needs to focus more on exploring the mechanism of gene body methylation and tumour invasion and development, seeking clearer and more definite pathophysiological mechanisms, and translating them into clinical applications to provide a more accurate judgement for tumour treatment and prognosis.

## Conclusion and perspective

The study of genome-wide methylation has led to the initial elucidation of the mechanism of gene body methylation occurrence and the regulation of gene expression. However, the specific role of gene body methylation in gene transcription is still widely debated. Gene body methylation suppresses gene expression by promoting chromatin densification [47] and interacting with functional elements in transcribed regions, such as alternative promoters, enhancers, TF binding sites, and repetitive elements [55]. However, numerous studies have shown that gene body methylation plays a positive role in transcription by suppressing spurious gene transcription, regulating alternative splicing, ensuring correct splicing and translation, and ensuring stable and orderly transcription. In addition, gene body methylation regulates transcription, while it is regulated by active transcription. Therefore, the generation of gene body methylation patterns and the specific molecular mechanisms of biological effects are still key questions that need to be solved and are future breakthrough directions in the field of gene body methylation.

In the field of oncology, current studies have shown that gene body methylation is essential for maintaining the stability of gene transcription and genomes. Tumour genomic hypomethylation can reduce chromosomal stability, disrupt genomic imprinting, and lead to abnormal tumour metabolism and differentiation, which are closely related to tumour occurrence, treatment, and prognosis. However, the mechanism of gene body hypomethylation in tumours is still unclear, and there are mainly two conjectures: (1) one conjecture suggests that tumour gene body hypomethylation is an active demethylation process, and TET family proteins, AICDA, and ELP3 are associated with the demethylation of methylated cytosines [126–128]. However, it was found that none of these genes were significantly higher in breast cancer cells than in normal breast epithelial cells [129]. (2) Another hypothesis holds that DNA methylation is passively lost during tumour replication and occurs mainly in late-replicating regions of the genome [130]. However, it has been shown that maladjustment of DNA repair mechanisms or DNA methyltransferases is not the main cause [131], so the cause of partial loss of tumour DNA methylation levels still needs to be further experimentally explored. In addition, Yen et al. found that gene bodies were completely methylated in two choriocarcinoma cell lines but hypomethylated in mesenchymal cells and placental cells [132], suggesting that tumour gene body hypomethylation is also not absolute and that more complex mechanisms may exist.

Current research on promoter DNA methylation is still just the tip of the DNA methylation iceberg. With the iterative update of DNA methylation sequencing technology and the decreasing cost of sequencing, research on DNA methylation will boom. The discovery of oxidative bisulfite sequencing (oxBS-Seq) in recent years has solved the previous problem of being unable to distinguish DNA methylation from hydroxymethylation, making the study of DNA methylation more accurate. With the availability of large amounts of sequencing data, the challenge ahead is to ensure the depth of sequence reads and the optimization of computational methods. By solving the above challenges, the mechanism and function of DNA methylation can be systematically elaborated and refined, and DNA methylation will represent a new generation of epigenetic biomarkers with unprecedented clinical value.

## Data Availability

Not applicable.

## Abbreviations

CGIs: CpG islands
DNMT: DNA methyltransferase
EB: Embryoid body
Phf6: Plant homologous structural domain finger protein 6
HP1: Heterochromatin protein 1
SRSF3: Serine- and arginine-rich splicing factor 3
TF: Transcription factors
EMT: Epithelial–mesenchymal transition
TNBC: Triple-negative breast cancer
DKO cells: HCT116 human colorectal cancer cells inactivated with DNMT1 and DNMT3B
REs: Repeat elements
HBx: Hepatitis B virus X
HDAC1: Histone deacetylase 1
PODXL: Podocalyxin-like protein 1
ITPKA: Inositol triphosphate 3-kinase A gene
HOTAIR: HOX transcript antisense RNA
miRNAs: MicroRNAs
FDA: Food and Drug Administration
GRA: 18β-glycyrrhetinic acid
LINE-1: Long interspersed nuclear elements 1
CRC: Colorectal cancer
TREX2: Triple repair nucleic acid exonuclease 2
AREG: Amphiregulin
EGFR: Epidermal growth factor receptor
oxBS-Seq: Oxidative bisulfites
